# Supplementary material for: Re-evaluating malarial retinopathy to improve its diagnostic accuracy in paediatric cerebral malaria: A retrospective study
Source: PLoS Med. 2025 Sep 15;22(9):e1004727. doi: 10.1371/journal.pmed.1004727 (PMC12445540; doi:10.1371/journal.pmed.1004727)
Supplement: S2 Analysis — Analysis required to produce tables and figures included in the supplementary information. Contains sensitivity analyses and analyses of spectrum bias. (PDF) [file pmed.1004727.s003.pdf]

Sensitivity analyses, post-hoc power calculations and evaluation of spectrum bias

Kyle J Wilson Alice Muiruri Liomba Karl B Seydel
Owen K Banda Christopher A Moxon Ian JC MacCormick
Simon P Harding Nicholas AV Beare Terrie E Taylor

1 Introduction

To assess the effect of missing data on our cohort, we first describe the characteristics of included participants and those with missing values for the index or reference tests, then perform worst- and best-case scenario sensitivity analyses.

Next, we explore spectrum bias within our cohort. Discussions of the inherent biases of autopsy cohort are found in the main article.

First though, we should read the data output from S1 Analysis.

```
# install and load packages necessary for analysis and displaying the data
packages <- c("readr", "tidyr", "readxl", "stringr", "testCompareR", "dplyr",
              "here", "tibble", "mice", "glmnet", "Matrix", "flextable",
              "ggplot2", "purrr")

for (pkg in packages) {
  if (!requireNamespace(pkg, quietly = TRUE)) {
    install.packages(pkg)
  }
  library(pkg, character.only = TRUE)
}

set.seed(0)

# complete cohort (with reference test result)
filename <- here("../AnonData/CompleteCohort.RData")
dat <- readRDS(filename) #%>% filter(complete.cases(true_cm))
```

```r
ref1 <- dat %>% filter(complete.cases(final_ret) &
                         complete.cases(true_cm)) %>% select(2:3)
ref1 <- summariseR(ref1)

filename <- here("../AnonData/OphthCohort.RData")
ophth <- readRDS(filename) %>% filter(complete.cases(true_cm))

ref2 <- ophth %>%
  filter(complete.cases(ophth_ret)) %>%
  select(ophth_ret, true_cm)
ref2 <- summariseR(ref2)
```

Then we produce the comparative table for the included and missing cohorts.

```r
make_summary <- function(df) {
  tibble::tibble(
    Age = sprintf("%.1f (%.1f - %.1f)", median(df$age, na.rm = TRUE),
                  min(df$age, na.rm = TRUE), max(df$age, na.rm = TRUE)),
    `No. Male (%)` = sprintf("%d (%.1f)", sum(df$sex, na.rm = TRUE),
                             mean(df$sex, na.rm = TRUE) * 100),
    `No. Fever (%)` = sprintf("%d (%.1f)", sum(df$hxfever, na.rm = TRUE),
                              mean(df$hxfever, na.rm = TRUE) * 100),
    `Fever Duration (h)` = sprintf("%.1f (%.1f - %.1f)",
                                   median(df$feverdur, na.rm = TRUE),
                                   min(df$feverdur, na.rm = TRUE),
                                   max(df$feverdur, na.rm = TRUE)),
    `Temperature (C)` = sprintf("%.1f (%.1f - %.1f)",
                                median(df$temp, na.rm = TRUE),
                                min(df$temp, na.rm = TRUE),
                                max(df$temp, na.rm = TRUE)),
    `Heart Rate (/min)` = sprintf("%.1f (%.1f - %.1f)",
                                  median(df$pulse, na.rm = TRUE),
                                  min(df$pulse, na.rm = TRUE),
                                  max(df$pulse, na.rm = TRUE)),
    `Resp. Rate (/min)` = sprintf("%.1f (%.1f - %.1f)",
                                  median(df$resp, na.rm = TRUE),
                                  min(df$resp, na.rm = TRUE),
                                  max(df$resp, na.rm = TRUE)),
    `Syst. BP (mmHg)` = sprintf("%.1f (%.1f - %.1f)",
                                median(df$bp, na.rm = TRUE),
                                min(df$bp, na.rm = TRUE),
```

```
                                max(df$bp, na.rm = TRUE)),
    `Weight (kg)` = sprintf("%.1f (%.1f - %.1f)",
                            median(df$wt, na.rm = TRUE),
                            min(df$wt, na.rm = TRUE),
                            max(df$wt, na.rm = TRUE)),
    `No. BCS 0 (%)` = sprintf("%d (%.1f)", sum(df$comasc == 0, na.rm = TRUE),
                              mean(df$comasc == 0, na.rm = TRUE) * 100),
    `No. BCS 1 (%)` = sprintf("%d (%.1f)", sum(df$comasc == 1, na.rm = TRUE),
                              mean(df$comasc == 1, na.rm = TRUE) * 100),
    `No. BCS 2 (%)` = sprintf("%d (%.1f)", sum(df$comasc == 2, na.rm = TRUE),
                              mean(df$comasc == 2, na.rm = TRUE) * 100),
    `No. BCS > 2 (%)` = sprintf("%d (%.1f)", sum(df$comasc > 2, na.rm = TRUE),
                                mean(df$comasc > 2, na.rm = TRUE) * 100),
    `CSF Opening Pressure (mmH2O)` = sprintf("%.1f (%.1f - %.1f)",
                                             median(df$opencsf, na.rm = TRUE),
                                             min(df$opencsf, na.rm = TRUE),
                                             max(df$opencsf, na.rm = TRUE)),
    `Time to Death (h)` = sprintf("%.1f (%.1f - %.1f)",
                                  median(df$death, na.rm = TRUE),
                                  min(df$death, na.rm = TRUE),
                                  max(df$death, na.rm = TRUE))
  )
}

included <- dat %>%
  filter(complete.cases(final_ret, true_cm)) %>%
  make_summary() %>% t()

excluded <- dat %>%
  filter(!complete.cases(final_ret, true_cm)) %>%
  make_summary() %>% t()

comparison <- bind_cols(Included = included[,1], Missing = excluded[,1]) %>%
  as.data.frame()

rownames(comparison) <- rownames(included)
header1 <- paste0("n = ", nrow(dat %>%
                                 filter(complete.cases(final_ret, true_cm))))
header2 <- paste0("n = ", nrow(dat %>%
                                 filter(!complete.cases(final_ret, true_cm))))

ft1 <- comparison %>% rownames_to_column(var = "Variable") %>%
```

```
  flextable() %>%
  add_header_row(top = FALSE, values = c("", header1, header2)) %>%
  merge_at(i = 1:2, j = 1, part = "header") %>%
  theme_booktabs() %>%
  align(align = "center", part = "all") %>%
  autofit()

ft1 #%>% save_as_docx(path = here("S4_Table.docx"))
```

Variable	Included<br>n = 84	Missing<br>n = 19
Age	29.0 (6.0 - 127.0)	48.0 (9.0 - 144.0)
No. Male (%)	46 (54.8)	12 (63.2)
No. Fever (%)	71 (85.5)	16 (84.2)
Fever Duration (h)	48.0 (2.0 - 504.0)	72.0 (7.0 - 336.0)
Temperature (C)	38.0 (32.2 - 42.1)	38.4 (34.5 - 40.6)
Heart Rate (/min)	150.0 (52.0 - 233.0)	128.0 (60.0 - 179.0)
Resp. Rate (/min)	46.0 (20.0 - 84.0)	45.0 (28.0 - 68.0)
Syst. BP (mmHg)	104.0 (70.0 - 175.0)	108.0 (60.0 - 130.0)
Weight (kg)	10.4 (4.0 - 31.0)	14.0 (7.5 - 26.0)
No. BCS 0 (%)	30 (35.7)	5 (31.2)
No. BCS 1 (%)	33 (39.3)	3 (18.8)
No. BCS 2 (%)	17 (20.2)	4 (25.0)
No. BCS > 2 (%)	4 (4.8)	4 (25.0)
CSF Opening Pressure (mmH2O)	190.0 (9.0 - 320.0)	140.0 (100.0 - 210.0)
Time to Death (h)	12.5 (0.0 - 168.0)	7.0 (0.0 - 71.0)

2 Sensitivity analyses

Here, we seek to check the effect of missing data on our result by re-running the `testCompareR` analysis assuming best- and worst-case scenarios for the missing data. Ideally, this should not affect the performance metrics too much, such that the point estimates fall within the

confidence intervals established during complete case analysis. Because we had <5% missing data in the reference test (n = 4), we restricted sensitivity analyses to the index test to maximise interpretability.

```
sensitivity_analysis <- function(ref_list, sens_list) {

  if(inherits(ref_list, "summariseR")) {

    metrics <- list(
    Sensitivity = list(ref = ref_list$acc[1, ], val = sens_list$acc[1, 1]),
    Specificity = list(ref = ref_list$acc[2, ], val = sens_list$acc[2, 1]),
    PPV = list(ref = ref_list$pv[1, ], val = sens_list$pv[1, 1]),
    NPV = list(ref = ref_list$pv[2, ], val = sens_list$pv[2, 1]),
    PLR = list(ref = ref_list$lr[1, ], val = sens_list$lr[1, 1]),
    NLR = list(ref = ref_list$lr[2, ], val = sens_list$lr[2, 1])
    )

    for (name in names(metrics)) {
      val <- metrics[[name]]$val
      lci <- metrics[[name]]$ref[3]
      uci <- metrics[[name]]$ref[4]

      if (val >= lci & val <= uci) {
        cat(paste0(name, " falls within CIs of CCA.\n"))
      } else {
        cat(paste0(name, " does not fall within CIs of CCA.\n"))
      }
      cat("  SA estimate: ", val, "\n  CCA CI: ", lci, " - ", uci, "\n")
    }

  }

}
```

2.1 Best-case scenario for missing data

Best-case scenario analysis for missing data in the context of diagnostic tests means assigning every positive result in the reference test as a true positive (index test positive) and every negative result in the reference test a true negative (index test negative).

2.1.1 Missing index test in complete cohort (n = 15)

```r
sens1 <- dat %>% filter(complete.cases(true_cm))
sens1$final_ret <- ifelse((is.na(sens1$final_ret) & sens1$true_cm == 1), 1,
                   ifelse((is.na(sens1$final_ret) & sens1$true_cm == 0), 0,
                          sens1$final_ret))

sens1 <- testCompareR::summariseR(sens1[,2:3])

sensitivity_analysis(ref1, sens1)
```

```
Sensitivity falls within CIs of CCA.
  SA estimate:  90.6
  CCA CI:  77.6  -  95.6
Specificity falls within CIs of CCA.
  SA estimate:  78.3
  CCA CI:  57.2  -  84.8
PPV falls within CIs of CCA.
  SA estimate:  82.8
  CCA CI:  71.2  -  87.9
NPV falls within CIs of CCA.
  SA estimate:  87.8
  CCA CI:  75.2  -  90.7
PLR falls within CIs of CCA.
  SA estimate:  4.2
  CCA CI:  2  -  5.7
NLR falls within CIs of CCA.
  SA estimate:  0.1
  CCA CI:  0.1  -  0.3
```

2.1.1 Missing index test in sub-analysis (ophthalmogist-graded assessments, n = 4)

```r
sens2 <- ophth
sens2$ophth_ret <- ifelse((is.na(sens2$ophth_ret) & sens2$true_cm == 1), 1,
                   ifelse((is.na(sens2$ophth_ret) & sens2$true_cm == 0), 0,
                          sens2$ophth_ret))

sens2 <- sens2 %>% select(ophth_ret, true_cm)
sens2 <- testCompareR::summariseR(sens2)

sensitivity_analysis(ref2, sens2)
```

```
Sensitivity falls within CIs of CCA.
```

```
  SA estimate:  94.4
  CCA CI:  81.7  -  98.7
Specificity falls within CIs of CCA.
  SA estimate:  78.6
  CCA CI:  56.8  -  88.8
PPV falls within CIs of CCA.
  SA estimate:  85
  CCA CI:  73.6  -  91.8
NPV falls within CIs of CCA.
  SA estimate:  91.7
  CCA CI:  80.6  -  95.8
PLR falls within CIs of CCA.
  SA estimate:  4.4
  CCA CI:  2.1  -  7.7
NLR falls within CIs of CCA.
  SA estimate:  0.1
  CCA CI:  0  -  0.2
```

2.2 Worst-case scenario for missing data

Worst-case scenario analysis for missing data in the context of diagnostic tests means assigning every positive result in the reference test as a false negative (index test negative) and every negative result in the reference test a false positive (index test positive).

2.2.1 Missing index test in complete cohort (n = 15)

```r
sens3 <- dat %>% filter(complete.cases(true_cm))
sens3$final_ret <- ifelse((is.na(sens3$final_ret) & sens3$true_cm == 1), 0,
                   ifelse((is.na(sens3$final_ret) & sens3$true_cm == 0), 1,
                          sens3$final_ret))

sens3 <- testCompareR::summariseR(sens3[,2:3])

sensitivity_analysis(ref1, sens3)
```

```
Sensitivity falls within CIs of CCA.
  SA estimate:  79.2
  CCA CI:  77.6  -  95.6
Specificity falls within CIs of CCA.
  SA estimate:  58.7
  CCA CI:  57.2  -  84.8
```

```
PPV does not fall within CIs of CCA.
  SA estimate:  68.9
  CCA CI:  71.2  -  87.9
NPV does not fall within CIs of CCA.
  SA estimate:  71.1
  CCA CI:  75.2  -  90.7
PLR does not fall within CIs of CCA.
  SA estimate:  1.9
  CCA CI:  2  -  5.7
NLR does not fall within CIs of CCA.
  SA estimate:  0.4
  CCA CI:  0.1  -  0.3
```

2.2.2 Missing index test in sub-analysis (ophthalmogist-graded assessments, n = 4)

```r
sens4 <- ophth
sens4$ophth_ret <- ifelse((is.na(sens4$ophth_ret) & sens4$true_cm == 1), 0,
                   ifelse((is.na(sens4$ophth_ret) & sens4$true_cm == 0), 1,
                          sens4$ophth_ret))

sens4 <- sens4 %>% select(ophth_ret, true_cm)
sens4 <- testCompareR::summariseR(sens4)

sensitivity_analysis(ref2, sens4)
```

```
Sensitivity falls within CIs of CCA.
  SA estimate:  91.7
  CCA CI:  81.7  -  98.7
Specificity falls within CIs of CCA.
  SA estimate:  67.9
  CCA CI:  56.8  -  88.8
PPV falls within CIs of CCA.
  SA estimate:  78.6
  CCA CI:  73.6  -  91.8
NPV falls within CIs of CCA.
  SA estimate:  86.4
  CCA CI:  80.6  -  95.8
PLR falls within CIs of CCA.
  SA estimate:  2.9
  CCA CI:  2.1  -  7.7
NLR falls within CIs of CCA.
```

```
SA estimate:  0.1
CCA CI:  0  -  0.2
```

3 Exploring spectrum bias

Spectrum bias is an inherent limitation of autopsy studies and this has been discussed in the main article. Unfortunately, there is no suitable validation cohort because autopsy studies in paediatric cerebral malaria are few. Using a model to predict cerebral parasite sequestration as the ground truth in cohorts which include survivors is possible, but is likely to encounter problems of reliability because models lack sufficiently high performance to be used as a reference test or rely on malarial retinopathy as one of the input variables, thus introducing circular logic when evaluating the effect of adjusting the definition of malarial retinopathy.

We can approximate the effects of spectrum bias within cohort, however, by using clinical parameters as estimators of disease severity. One parameter which might give a clue as to how disease severity affects our estimates of sensitivity and specificity is time to death. This relies on the assumption that children who are more sick are closer to death. This assumption may not always hold - for example, a child may receive more intervention, there may be a higher level of care available to that child, or they may have a complication more amenable to intervention. All of these scenarios are independent of disease severity, but may influence time to death. It is worth bearing this in mind as we conduct the next analysis.

The first analysis we conducted to explore spectrum bias stratified children by time to death and selected children from the bottom quartile and top quartile. Because this reduces the already small sample size we used bootstrapping from these cohorts to create simulated data sets of n=60 (sub-analysis sample size) and n=157 (adequately powered sample).

With these data sets we can then look for differences in sensitivity and specificity of malarial retinopathy in the less severe and more severe groups. We can also evaluate the effect of adjusting the definition of malarial retinopathy in less severe and more severe group.

```
demo <- read_xlsx(here("../AnonData/S4_Data.xlsx"))
lq <- quantile(demo$death, 0.25)
uq <- quantile(demo$death, 0.75)

ophth <- ophth %>% left_join(demo %>% select(mp_number, death),
                                   join_by(mp_number == mp_number))

results <- replicate(n = 500, expr = {
    boot_sample <- ophth %>%
    filter(complete.cases(ophth_ret) & complete.cases(true_cm) &
             complete.cases(recoded)) %>%
    select(ophth_ret, recoded, true_cm, death) %>%
```

```
    slice_sample(n = 60, replace = TRUE)

    boot_sample_severe <- boot_sample %>% filter(death <= lq)
    boot_sample_mod <- boot_sample %>% filter(death >= uq)

    list(severe = testCompareR::compareR(test1 = boot_sample_severe$ophth_ret,
                              test2 = boot_sample_severe$recoded,
                              gold = boot_sample_severe$true_cm),
         moderate = testCompareR::compareR(test1 = boot_sample_mod$ophth_ret,
                              test2 = boot_sample_mod$recoded,
                              gold = boot_sample_mod$true_cm))},
    simplify = FALSE)

results_powered <- replicate(n = 500, expr = {
    boot_sample <- ophth %>%
    filter(complete.cases(ophth_ret) & complete.cases(true_cm) &
             complete.cases(recoded)) %>%
    select(ophth_ret, recoded, true_cm, death) %>%
    slice_sample(n = 157, replace = TRUE)

    boot_sample_severe <- boot_sample %>% filter(death <= lq)
    boot_sample_mod <- boot_sample %>% filter(death >= uq)

    list(severe = testCompareR::compareR(test1 = boot_sample_severe$ophth_ret,
                              test2 = boot_sample_severe$recoded,
                              gold = boot_sample_severe$true_cm),
         moderate = testCompareR::compareR(test1 = boot_sample_mod$ophth_ret,
                              test2 = boot_sample_mod$recoded,
                              gold = boot_sample_mod$true_cm))},
    simplify = FALSE)
```

We can summarise the results in a table by taking the mean across all 500 repititions for each of the models.

```
extract_metrics <- function(x) {
  tryCatch({
    tibble(
      sev_sens1 = x$severe$acc$accuracies$`Test 1`[1, 1],
      sev_spec1 = x$severe$acc$accuracies$`Test 1`[2, 1],
      sev_ppv1  = x$severe$pv$predictive.values$`Test 1`[1, 1],
      sev_npv1  = x$severe$pv$predictive.values$`Test 1`[2, 1],
      sev_plr1  = x$severe$lr$likelihood.ratios$`Test 1`[1, 1],
```

```r
      sev_nlr1  = x$severe$lr$likelihood.ratios$`Test 1`[2, 1],

      sev_sens2 = x$severe$acc$accuracies$`Test 2`[1, 1],
      sev_spec2 = x$severe$acc$accuracies$`Test 2`[2, 1],
      sev_ppv2  = x$severe$pv$predictive.values$`Test 2`[1, 1],
      sev_npv2  = x$severe$pv$predictive.values$`Test 2`[2, 1],
      sev_plr2  = x$severe$lr$likelihood.ratios$`Test 2`[1, 1],
      sev_nlr2  = x$severe$lr$likelihood.ratios$`Test 2`[2, 1],

      mod_sens1 = x$moderate$acc$accuracies$`Test 1`[1, 1],
      mod_spec1 = x$moderate$acc$accuracies$`Test 1`[2, 1],
      mod_ppv1  = x$moderate$pv$predictive.values$`Test 1`[1, 1],
      mod_npv1  = x$moderate$pv$predictive.values$`Test 1`[2, 1],
      mod_plr1  = x$moderate$lr$likelihood.ratios$`Test 1`[1, 1],
      mod_nlr1  = x$moderate$lr$likelihood.ratios$`Test 1`[2, 1],

      mod_sens2 = x$moderate$acc$accuracies$`Test 2`[1, 1],
      mod_spec2 = x$moderate$acc$accuracies$`Test 2`[2, 1],
      mod_ppv2  = x$moderate$pv$predictive.values$`Test 2`[1, 1],
      mod_npv2  = x$moderate$pv$predictive.values$`Test 2`[2, 1],
      mod_plr2  = x$moderate$lr$likelihood.ratios$`Test 2`[1, 1],
      mod_nlr2  = x$moderate$lr$likelihood.ratios$`Test 2`[2, 1]
    )
  }, error = function(e) NULL)
}

# Apply to all results
summary_df <- map_dfr(results, extract_metrics)
summary_df <- summary_df %>%
  mutate(across(everything(), ~replace(.x, is.infinite(.x), NA)))

# Mean summary
summary_means <- summary_df %>%
  summarise(across(everything(), ~round(mean(.x, na.rm = TRUE), digits = 1)))

summary_table <- summary_means %>%
  # Convert wide to long format
  pivot_longer(cols = everything(),
               names_to = "metric_group",
               values_to = "value") %>%
  # Separate components
  extract(metric_group, into = c("group", "metric", "test"),
```

```
         regex = "(sev|mod)_(.*?)([12])") %>%
  mutate(
    group = recode(group, sev = "Severe", mod = "Moderate"),
    test = paste0("Test ", test),
    metric = recode(metric,
                    sens = "Sensitivity",
                    spec = "Specificity",
                    ppv  = "PPV",
                    npv  = "NPV",
                    plr  = "PLR",
                    nlr  = "NLR")
  ) %>%
  # Create a combined column name for columns
  unite("group_test", group, test, sep = " - ") %>%
  pivot_wider(names_from = group_test, values_from = value) %>%
  # Optional: reorder rows
  arrange(factor(metric, levels = c("Sensitivity", "Specificity", "PPV", "NPV", "PLR", "NLR"

ft1 <- flextable(summary_table) %>%
  autofit() %>%
  align(align = "center", part = "all")

ft1
```

metric	Severe – Test 1	Severe – Test 2	Moderate – Test 1	Moderate – Test 2
Sensitivity	100.0	100.0	89.2	89.2
Specificity	51.1	87.8	89.6	89.6
PPV	64.4	88.0	89.8	89.8
NPV	100.0	100.0	88.8	88.8
PLR	2.4	6.4	6.2	6.2
NLR	0.0	0.0	0.1	0.1

S3 Table 1. Analysis of spectrum bias. *Comparison of the mean of all bootstrapped values (n=60) for diagnostic accuracies for children who are severely sick (time to death in bottom quartile) and those who are moderately sick (time to death in upper quartile). Test 1 represents the original definition of malarial retinopathy. Test 2 represents the definition in which 1-5 haemorrhages in one eye only is insufficient to predict cerebral parasite sequestration. PPV -*

positive predictive value; NPV - negative predictive value; PLR - positive likelihood ratio; NLR - negative likelihood ratio.

```
summary_df <- map_dfr(results_powered, extract_metrics)
summary_df <- summary_df %>%
  mutate(across(everything(), ~replace(.x, is.infinite(.x), NA)))

# Mean summary
summary_means <- summary_df %>%
  summarise(across(everything(), ~round(mean(.x, na.rm = TRUE), digits = 1)))

summary_table <- summary_means %>%
  # Convert wide to long format
  pivot_longer(cols = everything(),
               names_to = "metric_group",
               values_to = "value") %>%
  # Separate components
  extract(metric_group, into = c("group", "metric", "test"),
          regex = "(sev|mod)_(.*?)([12])") %>%
  mutate(
    group = recode(group, sev = "Severe", mod = "Moderate"),
    test = paste0("Test ", test),
    metric = recode(metric,
                    sens = "Sensitivity",
                    spec = "Specificity",
                    ppv  = "PPV",
                    npv  = "NPV",
                    plr  = "PLR",
                    nlr  = "NLR")
  ) %>%
  # Create a combined column name for columns
  unite("group_test", group, test, sep = " - ") %>%
  pivot_wider(names_from = group_test, values_from = value) %>%
  # Optional: reorder rows
  arrange(factor(metric, levels = c("Sensitivity", "Specificity", "PPV", "NPV", "PLR", "NLR"

ft2 <- flextable(summary_table) %>%
  autofit() %>%
  align(align = "center", part = "all")

ft2
```

metric	Severe – Test 1	Severe – Test 2	Moderate – Test 1	Moderate – Test 2
Sensitivity	100.0	100.0	88.9	88.9
Specificity	50.5	88.1	89.2	89.2
PPV	64.1	88.0	89.1	89.1
NPV	100.0	100.0	89.0	89.0
PLR	2.1	10.2	10.0	10.0
NLR	0.0	0.0	0.1	0.1

S3 Table 2. Analysis of spectrum bias. *Comparison of the mean of all bootstrapped values (n=157) for diagnostic accuracies for children who are severely sick (time to death in bottom quartile) and those who are moderately sick (time to death in upper quartile). Test 1 represents the original definition of malarial retinopathy. Test 2 represents the definition in which 1-5 haemorrhages in one eye only is insufficient to predict cerebral parasite sequestration. PPV - positive predictive value; NPV - negative predictive value; PLR - positive likelihood ratio; NLR - negative likelihood ratio.*

Here we can see that changing the definition of malarial retinopathy such that 1-5 haemorrhages in one eye only is not considered predictive of cerebral parasite sequestration appears to only affect children in the 'severe' group (that is, those who died most quickly after admission). This suggests that this change in the definition is most useful in severe cases. It should be noted, though, that this change didn't appear to detrimentally affect less severe children in this cohort. Given that being declared retinopathy negative should increase clinician vigilance, the safest thing for patients in whom the sole sign of malarial retinopathy is 1-5 haemorrhages in one eye only is to classify them as 'at risk' of misdiagnosis and consider alternatives quickly if there is limited response to treatment.

Another method to evaluate the effects of clinical parameters (surrogates for disease severity) on test performance is to treat it as a classification problem and use logistic regression to predict the binary outcome of the response variable (reference test) from predictor variables including the index test (binary) and clinical variables. Using the LASSO regularisation method allows us to assess whether clinical variables or the interaction between the index test and clinical variables are important for prediction accuracy. LASSO regression may penalise the index test, reducing the model coefficient to zero. To avoid this, we can set the penalty for this variable to zero and see whether any other clinical variables included in the model exert an influence on the prediction over and above the effect of the index test.

Because construction of the model matrix requires complete data, we used multiple imputation by chained equations to produce 10 data sets for LASSO logistic regression and evaluated how frequently predictors were retained following LASSO penalisation and the mean effect size. We ran the LASSO logistic regression using the original cohort (n=84).

```r
vars <- c("true_cm", "final_ret", "age", "sex", "feverdur", "temp", "pulse",
          "resp", "bp", "wt", "comasc", "death")

dat <- dat %>% select(all_of(vars))

imputed <- mice(dat, m = 10, seed = 0, printFlag = FALSE)

coef_list <- list()

for (i in 1:10) {

  dat_i <- complete(imputed, i)

  x <- model.matrix(true_cm ~ final_ret *
                      (age + sex + feverdur + temp + pulse + resp +
                       bp + wt + comasc + death), data = dat_i)[, -1]
  y <- dat_i$true_cm

  # do not penalise index test
  penalty_factors <- rep(1, ncol(x))
  penalty_factors[which(colnames(x) == "final_ret")] <- 0

  fit <- cv.glmnet(x, y, alpha = 1, family = "binomial",
                   penalty.factor = penalty_factors)
  coefs <- coef(fit, s = "lambda.1se")

  coef_list[[i]] <- as.vector(coefs)
  names(coef_list[[i]]) <- rownames(coefs)

}

coef_df <- do.call(rbind, coef_list) %>% as.data.frame()
coef_df <- coef_df[, colSums(coef_df != 0) > 0]

summary_df <- coef_df %>%
  pivot_longer(cols = everything(), names_to = "variable", values_to = "coef") %>%
  group_by(variable) %>%
  summarise(
    times_selected = sum(coef != 0),
    mean_coef = mean(coef[coef != 0]),
    sd_coef = sd(coef[coef != 0]),
    .groups = "drop"
```

```r
  ) %>%
  arrange(desc(times_selected), desc(abs(mean_coef)))

summary_df
```

```
# A tibble: 2 x 4
  variable    times_selected mean_coef sd_coef
  <chr>                <int>     <dbl>   <dbl>
1 final_ret               10      3.18  0.169
2 (Intercept)             10     -1.88  0.0999
```

Here we have used LASSO as a type of sensitivity analysis for spectrum bias within our cohort. Here, we can see that the coefficients of all clinical variables are reduced to zero by the LASSO penalty in all imputations, suggesting that they don't exert a strong effect on the outcome of the reference test or the index test. It should be noted that frequency is a simple heuristic measure of robustness.

Overall, the exploration of spectrum bias suggests that clinical features are unlikely to influence the utility of the index test in an autopsy cohort, but the re-classification of 1-5 haemorrhages in a single eye only may be more important in severe cases, where retinopathy, when present is likely to be severe. Models which accurately predict cerebral parasite sequestration from clinical data are necessary to further validate this finding in cohorts which include survivors.

```r
sessionInfo()
```

```
R version 4.4.0 (2024-04-24)
Platform: aarch64-apple-darwin20
Running under: macOS Ventura 13.5.1

Matrix products: default
BLAS:   /Library/Frameworks/R.framework/Versions/4.4-arm64/Resources/lib/libRblas.0.dylib
LAPACK: /Library/Frameworks/R.framework/Versions/4.4-arm64/Resources/lib/libRlapack.dylib;  [illegible]

locale:
[1] en_US.UTF-8/en_US.UTF-8/en_US.UTF-8/C/en_US.UTF-8/en_US.UTF-8

time zone: Europe/London
tzcode source: internal

attached base packages:
[1] stats     graphics  grDevices utils     datasets  methods   base
```

```
other attached packages:
 [1] purrr_1.1.0        ggplot2_3.5.2      flextable_0.9.9    glmnet_4.1-10
 [5] Matrix_1.7-3       mice_3.18.0        tibble_3.3.0       here_1.0.1
 [9] dplyr_1.1.4        testCompareR_1.1.1 stringr_1.5.1      readxl_1.4.5
[13] tidyr_1.3.1        readr_2.1.5

loaded via a namespace (and not attached):
 [1] gtable_0.3.6           shape_1.4.6.1          xfun_0.52
 [4] lattice_0.22-7         tzdb_0.5.0             vctrs_0.6.5
 [7] tools_4.4.0            Rdpack_2.6.4           generics_0.1.4
[10] pan_1.9                pkgconfig_2.0.3        jomo_2.7-6
[13] data.table_1.17.8      RColorBrewer_1.1-3     uuid_1.2-1
[16] lifecycle_1.0.4        farver_2.1.2           compiler_4.4.0
[19] textshaping_1.0.1      codetools_0.2-20       fontquiver_0.2.1
[22] fontLiberation_0.1.0   htmltools_0.5.8.1      yaml_2.3.10
[25] pillar_1.11.0          nloptr_2.2.1           MASS_7.3-65
[28] openssl_2.3.3          reformulas_0.4.1       iterators_1.0.14
[31] rpart_4.1.24           boot_1.3-31            foreach_1.5.2
[34] mitml_0.4-5            nlme_3.1-168           fontBitstreamVera_0.1.1
[37] zip_2.3.3              tidyselect_1.2.1       digest_0.6.37
[40] stringi_1.8.7          splines_4.4.0          rprojroot_2.1.0
[43] fastmap_1.2.0          grid_4.4.0             cli_3.6.5
[46] magrittr_2.0.3         utf8_1.2.6             survival_3.8-3
[49] broom_1.0.8            withr_3.0.2            scales_1.4.0
[52] gdtools_0.4.2          backports_1.5.0        officer_0.6.10
[55] rmarkdown_2.29         nnet_7.3-20            lme4_1.1-37
[58] cellranger_1.1.0       ragg_1.4.0             askpass_1.2.1
[61] hms_1.1.3              evaluate_1.0.4         knitr_1.50
[64] rbibutils_2.3          rlang_1.1.6            Rcpp_1.1.0
[67] glue_1.8.0             xml2_1.3.8             rstudioapi_0.17.1
[70] minqa_1.2.8            jsonlite_2.0.0         R6_2.6.1
[73] systemfonts_1.2.3
```
