# Supplementary material for: Re-evaluating malarial retinopathy to improve its diagnostic accuracy in paediatric cerebral malaria: A retrospective study
Source: PLoS Med. 2025 Sep 15;22(9):e1004727. doi: 10.1371/journal.pmed.1004727 (PMC12445540; doi:10.1371/journal.pmed.1004727)
Supplement: S3 Analysis — Analysis required to verify inter-rater reliability of grading ophthalmic images. (PDF) [file pmed.1004727.s004.pdf]

Inter-rater and inter-method concordance of malarial retinopathy grading

Kyle J Wilson Alice Muiruri Liomba Karl B Seydel
Owen K Banda Christopher A Moxon Ian JC MacCormick
Simon P Harding Nicholas AV Beare Terrie E Taylor

1 Introduction

Indirect ophthalmoscopy is the gold standard test for malarial retinopathy. However, it is impractical to blind an ophthalmologist or clinician from coma status and parasitaemia in a high acuity clinical setting. This could potentially be a source of unmeasured bias.

To demonstrate that grading is consistent, two assessors graded 80 retinal photos of 80 eyes from 41 participants over the same study period. One assessor (KJW) is an ophthalmologist experienced in the assessment of malarial retinopathy. The second assessor (OKB) is an optometrist more recently trained in the assessment of malarial retinopathy. By including graders with differing levels of experience we also demonstrate that agreement is robust to level of training.

2 Analysis

2.1 Inter-rater concordance from retinal images

We first show agreement for malarial retinopathy status (positive or negative) and then for the individual signs of malarial retinopathy. First, we prepare the data.

```r
# install and load packages necessary for analysis and displaying the data
packages <- c("readr", "tidyr", "readxl", "stringr", "dplyr", "here", "vcd")

for (pkg in packages) {
  if (!requireNamespace(pkg, quietly = TRUE)) {
    install.packages(pkg)
  }
```

```
  library(pkg, character.only = TRUE)
}

set.seed(0)

dual <- read_xlsx(here("../AnonData/S5_Data.xlsx"))
dual <- dual %>% mutate(across(2:11, ~na_if(.x, -9)))

dual <- dual %>%
  rowwise() %>%
  mutate(status_ob = case_when(
    all(c_across(2:6) == 0, na.rm = FALSE) ~ 0,
    all(is.na(c_across(2:6)) | c_across(2:6) == 0) ~ NA_real_,
    any(c_across(2:6) != 0, na.rm = TRUE) ~ 1),
    status_kw = case_when(
    all(c_across(7:11) == 0, na.rm = FALSE) ~ 0,
    all(is.na(c_across(7:11)) | c_across(7:11) == 0) ~ NA_real_,
    any(c_across(7:11) != 0, na.rm = TRUE) ~ 1)
  ) %>%
  ungroup()
```

Next, we compare grading of the presence or absence of MR.

```
tab <- table(dual$status_ob, dual$status_kw)
res.k <- vcd::Kappa(tab)
ci.k <- confint(res.k)
cat("MR status: ", round(res.k$Unweighted["value"], 3),
    " (", round(ci.k[2,1], 3), " - ", round(ci.k[2,2], 3), ")\n\n", sep = "")
```

```
MR status: 0.63 (0.365 - 0.894)
```

Then we look at individual features of MR.

```
features <- c("ahaem", "acfa", "awhitcmac", "apw", "avc")
feature_names <- c(
  ahaem = "Haemorrhages",
  acfa = "Foveal whitening",
  awhitcmac = "Macular whitening",
  apw = "Peripheral whitening",
  avc = "Vessel change"
)
```

```
for (i in seq_along(features)) {
  feat <- features[i]
  name <- feature_names[feat]
  tab <- table(dual[[paste0(feat, "_ob")]], dual[[paste0(feat, "_kw")]])
  res.k <- vcd::Kappa(tab, "Fleiss-Cohen")
  ci.k <- confint(res.k)
  cat(name, ": ",
      round(res.k$Weighted["value"], 3),
      " (", round(ci.k[2,1], 3), " - ", round(ci.k[2,2], 3),
      ")\n\n", sep = "")
}
```

```
Haemorrhages: 0.977 (0.959 - 0.994)

Foveal whitening: 0.7 (0.589 - 0.811)

Macular whitening: 0.672 (0.527 - 0.817)

Peripheral whitening: 0.482 (0.286 - 0.679)

Vessel change: 0.525 (0.337 - 0.713)
```

There is substantial agreement (according to Landis and Koch, 1977) in assessment of retinopathy status. There is near perfect agreement in assessment of haemorrhages between the two graders from photos. There is substantial agreement of foveal whitening and macular whitening. There is moderate agreement in both peripheral whitening and vessel change.

These results corroborate findings from Beare et al, 2002 that agreement is good between graders and extend these findings to demonstrate good agreement between graders with different levels of experience and training.

2.2 Inter-method reliability between indirect ophthalmoscopy and image grading

Next, we wanted to compare the data from the photo grading with the findings from clinical examination. First, we prepared the data.

```
rtd <- read.csv(here("../AnonData/S6_Data.csv"))

rtd <- rtd %>% filter(mp_number %in% dual$mp_number)
rtd <- rtd %>%
  rowwise() %>%
```

```r
  mutate(status = case_when(
    all(c_across(2:6) == 0, na.rm = FALSE) ~ 0,
    all(is.na(c_across(2:6)) | c_across(2:6) == 0) ~ NA_real_,
    any(c_across(2:6) != 0, na.rm = TRUE) ~ 1)
  ) %>%
  ungroup()

rtd <- left_join(rtd, dual, join_by(mp_number == mp_number))
```

Now, we can conduct the analysis. For this analysis we used the ophthalmologist grading (KJW) and compared to graded ophthalmologist assessment. Once again, first we look at presence and absence of MR.

```r
tab <- table(rtd$status, rtd$status_kw)
res.k <- vcd::Kappa(tab)
ci.k <- confint(res.k)
cat("MR status: ", round(res.k$Unweighted["value"], 3),
    " (", round(ci.k[2,1], 3), " - ", round(ci.k[2,2], 3), ")\n\n", sep = "")
```

```
MR status: 0.839 (0.622 - 1)
```

Then we look at the individual features of MR.

```r
for (i in seq_along(features)) {
  feat <- features[i]
  name <- feature_names[feat]
  tab <- table(rtd[[feat]], rtd[[paste0(feat, "_kw")]])
  res.k <- vcd::Kappa(tab, "Fleiss-Cohen")
  ci.k <- confint(res.k)
  cat(name, ": ",
      round(res.k$Weighted["value"], 3),
      " (", round(ci.k[2,1], 3), " - ", round(ci.k[2,2], 3),
      ")\n\n", sep = "")
}
```

```
Haemorrhages: 0.767 (0.623 - 0.911)

Foveal whitening: 0.685 (0.523 - 0.846)

Macular whitening: 0.776 (0.64 - 0.912)
```

```
Peripheral whitening: 0.578 (0.364 - 0.793)

Vessel change: 0.348 (0.148 - 0.547)
```

Here, we can see that while agreement is generally good, agreement in haemorrhage grading is worse than in photos. Peripheral findings also have lower agreement than would be ideal. Because we are interested in retinal haemorrhage status, we next interrogated whether the differences tend to go in one direction or the other.

To do this, we simply subtracted the score from the examination from the grading score from stored images, then calculated the mean. If there was a directional effect this would result in a negative number (undergrading from the photos) or positive number (overgrading from the photos). The significance of this directional effect was assessed using the Mann-Whitney-U test for non-parametric data.

```r
p_values <- numeric(length(features))
mean_diffs <- numeric(length(features))

for (i in seq_along(features)) {
  feat <- features[i]
  photo_col <- paste0(feat, "_kw")
  exam_col <- feat

  test <- wilcox.test(rtd[[photo_col]], rtd[[exam_col]], paired = TRUE)

  mean_diffs[i] <- mean(rtd[[photo_col]] - rtd[[exam_col]], na.rm = TRUE)
  p_values[i] <- test$p.value
}

adjusted_p <- p.adjust(p_values, method = "holm")

for (i in seq_along(features)) {
  feat <- features[i]
  label <- feature_names[[feat]]
  mean_diff <- mean_diffs[i]
  adj_p <- adjusted_p[i]

  grade <- if (mean_diff < 0) "undergraded" else "overgraded"
  miss <- if (mean_diff < 0) "missed" else "recognised erroneously"

  direction <- if (adj_p < 0.05) "This is statistically significant."
  else "This is not statistically significant."
```

```
  if(feat %in% c("ahaem", "acfa", "awhitcmac")) {
    cat(
    "Feature: ", label, "\n",
    "Mean difference: ", round(mean_diff, 3), "\n",
    "Adjusted p-value: ", signif(adj_p, 3), "\n",
    "Interpretation: Approximately 1 in ", abs(round(1 / mean_diff, 0)),
    " eyes are ", grade, " by 1 level. \n", direction, "\n\n", sep = ""
    )
  } else {
    cat(
    "Feature: ", label, "\n",
    "Mean difference: ", round(mean_diff, 3), "\n",
    "Adjusted p-value: ", signif(adj_p, 3), "\n",
    "Interpretation: ", label, " is ", miss, " in approximately 1 in ",
    abs(round(1 / mean_diff, 0)), " eyes. \n", direction, "\n\n", sep = ""
    )
  }

}
```

```
Feature: Haemorrhages
Mean difference: -0.467
Adjusted p-value: 0.00126
Interpretation: Approximately 1 in 2 eyes are undergraded by 1 level.
This is statistically significant.

Feature: Foveal whitening
Mean difference: 0
Adjusted p-value: 0.972
Interpretation: Approximately 1 in Inf eyes are overgraded by 1 level.
This is not statistically significant.

Feature: Macular whitening
Mean difference: -0.164
Adjusted p-value: 0.127
Interpretation: Approximately 1 in 6 eyes are undergraded by 1 level.
This is not statistically significant.

Feature: Peripheral whitening
Mean difference: -0.123
Adjusted p-value: 0.118
```

```
Interpretation: Peripheral whitening is missed in approximately 1 in 8 eyes.
This is not statistically significant.

Feature: Vessel change
Mean difference: -0.276
Adjusted p-value: 0.0015
Interpretation: Vessel change is missed in approximately 1 in 4 eyes.
This is statistically significant.
```

3 Conclusions

Taken together, these findings show that, while there is considerable agreement between graders (including graders of different training levels), malarial retinopathy grading by photos alone risks systematically undergrading haemorrhages and vessel change (a peripheral finding).

```r
sessionInfo()
```

```
R version 4.4.0 (2024-04-24)
Platform: aarch64-apple-darwin20
Running under: macOS Ventura 13.5.1

Matrix products: default
BLAS:   /Library/Frameworks/R.framework/Versions/4.4-arm64/Resources/lib/libRblas.0.dylib
LAPACK: /Library/Frameworks/R.framework/Versions/4.4-arm64/Resources/lib/libRlapack.dylib;  [illegible]

locale:
[1] en_US.UTF-8/en_US.UTF-8/en_US.UTF-8/C/en_US.UTF-8/en_US.UTF-8

time zone: Europe/London
tzcode source: internal

attached base packages:
[1] grid      stats     graphics  grDevices utils     datasets  methods
[8] base

other attached packages:
[1] vcd_1.4-13    here_1.0.1    dplyr_1.1.4   stringr_1.5.1 readxl_1.4.5
[6] tidyr_1.3.1   readr_2.1.5

loaded via a namespace (and not attached):
 [1] jsonlite_2.0.0    compiler_4.4.0    tidyselect_1.2.1  yaml_2.3.10
```

```
 [5] fastmap_1.2.0      lattice_0.22-7     R6_2.6.1           generics_0.1.4
 [9] lmtest_0.9-40      knitr_1.50         MASS_7.3-65        tibble_3.3.0
[13] rprojroot_2.1.0    pillar_1.11.0      tzdb_0.5.0         rlang_1.1.6
[17] stringi_1.8.7      xfun_0.52          cli_3.6.5          withr_3.0.2
[21] magrittr_2.0.3     digest_0.6.37      rstudioapi_0.17.1 hms_1.1.3
[25] lifecycle_1.0.4    vctrs_0.6.5        evaluate_1.0.4     glue_1.8.0
[29] cellranger_1.1.0   zoo_1.8-14         colorspace_2.1-1   rmarkdown_2.29
[33] purrr_1.1.0        tools_4.4.0        pkgconfig_2.0.3    htmltools_0.5.8.1
```
