# Supplementary material for: Re-evaluating malarial retinopathy to improve its diagnostic accuracy in paediatric cerebral malaria: A retrospective study
Source: PLoS Med. 2025 Sep 15;22(9):e1004727. doi: 10.1371/journal.pmed.1004727 (PMC12445540; doi:10.1371/journal.pmed.1004727)
Supplement: S1 Table — (DOCX) [file pmed.1004727.s005.docx]

| Variable | Included | Missing |
| --- | --- | --- |
|  | n = 84 | n = 19 |
| Age | 29.0 (6.0 - 127.0) | 48.0 (9.0 - 144.0) |
| No. Male (%) | 46 (54.8) | 12 (63.2) |
| No. Fever (%) | 71 (85.5) | 16 (84.2) |
| Fever Duration (h) | 48.0 (2.0 - 504.0) | 72.0 (7.0 - 336.0) |
| Temperature (C) | 38.0 (32.2 - 42.1) | 38.4 (34.5 - 40.6) |
| Heart Rate (/min) | 150.0 (52.0 - 233.0) | 128.0 (60.0 - 179.0) |
| Resp. Rate (/min) | 46.0 (20.0 - 84.0) | 45.0 (28.0 - 68.0) |
| Syst. BP (mmHg) | 104.0 (70.0 - 175.0) | 108.0 (60.0 - 130.0) |
| Weight (kg) | 10.4 (4.0 - 31.0) | 14.0 (7.5 - 26.0) |
| No. BCS 0 (%) | 30 (35.7) | 5 (31.2) |
| No. BCS 1 (%) | 33 (39.3) | 3 (18.8) |
| No. BCS 2 (%) | 17 (20.2) | 4 (25.0) |
| No. BCS > 2 (%) | 4 (4.8) | 4 (25.0) |
| CSF Opening Pressure (mmH2O) | 190.0 (9.0 - 320.0) | 140.0 (100.0 - 210.0) |
| Time to Death (h) | 12.5 (0.0 - 168.0) | 7.0 (0.0 - 71.0) |
