# Supplementary material for: Re-evaluating malarial retinopathy to improve its diagnostic accuracy in paediatric cerebral malaria: A retrospective study
Source: PLoS Med. 2025 Sep 15;22(9):e1004727. doi: 10.1371/journal.pmed.1004727 (PMC12445540; doi:10.1371/journal.pmed.1004727)
Supplement: Attachment — Submitted filename: 20250519_MRSens_ReviewerResponses_v1.0.docx [file pmed.1004727.s014.docx]

We thank the reviewers for their positive feedback and helpful comments on how this manuscript can be improved. Find below point-by-point response addressing all of these comments with references to where changes have been made in the manuscript. We think that these changes have unquestionably improved the manuscript. We are very willing to enter any further discussion with the Reviewers and Editorial Team in order to make this manuscript as useful and applicable to the clinical and scientific communities as possible.

I am sure you will agree that, with the substantial changes made, this paper is greatly improved. Thank you for your time and effort!

**Comments from the academic editor:**
Please discuss how you would suggest to increase the uptake of retinal examination in busy clinical settings where malaria is endemic, or whether you think that retinoscopy is exclusively a research tool.

*Good suggestion, thank you. We have added a line to the discussion and a couple of references which discuss this further. See page 8, line 267 – 271.*

**Comments from the reviewers:**

**Reviewer #1:**

This research extends an earlier paediatric autopsy study to further investigate the value of malarial retinopathy (MR) in diagnosing cerebral malaria (CM). A retrospective analysis was performed on autopsy data (the reference standard for diagnosing CM) and clinical diagnostic data for both MR and CM. The MR clinical diagnoses were performed by either ophthalmologists or clinicians trained in retinal examination. The authors found clinical MR diagnoses generally had good sensitivity and specificity to predict CM, however accuracy was improved by using only the ophthalmologist-diagnosed MR data and a higher threshold for MR diagnosis.

This is a well-written paper that effectively contextualises and establishes the importance of the research presented. While there are some opportunities for clarifying the background to the study, the methods used, and the results obtained, these parts of the manuscript are broadly cogent and clear. Indeed, I particularly want to commend the authors for including R markdown annotated code, which I found very helpful in understanding their analyses. That said, I have more substantial concerns about the content and structure of the Discussion and suggest a more comprehensive rethink and rewrite.

Detailed feedback on the manuscript is provided below. My primary focus has been methodological, but I have made a few broader points as well.  All items are major unless indicated otherwise.

1.      Suggest including 95% confidence intervals for estimated statistics (i.e., sensitivity and specificity) in the abstract.

*Done, see page 2, line 40 – 43.*

2.      Suggest the authors briefly explain why the focus of this research is paediatric. The authors state that children bear most of the burden of disease of malaria, but is it also that children are particularly at risk of CM? Or something else entirely?

*Done, see page 3, line 76 – 78.*

3.      [MINOR] Consider indicating in the title that the population is paediatric.

*Done. This is actually a very important point. Malarial retinopathy does not represent cerebral disease in adults in the same way that it does in children. See title.*

4.      Suggest avoiding words like "significant" and "significance" unless discussing statistical significance, accompanied by referenced or actual statistical results. Reconsider wording like "Malarial retinopathy (MR) has diagnostic and prognostic significance in CM." and "This study confirms that a significant proportion of patients apparently dying of WHO-defined CM have a non-malarial cause of death...".

*Done, avoided throughout.*

5.      If I am understanding correctly "(cerebral) (parasite) sequestration" is effectively interchangeable in the context of this paper with both "histopathologically-proven CM" and "CM positive (by autopsy)" i.e., this study's reference standard. I found this quite confusing on my first read through - particularly in Figure 1 where the "CM" abbreviation is defined but "sequestration" is used instead. Suggest making these definitions clear early and, as much as possible, sticking to one wording that is readily understandable.

*We have two primary definitions, the clinical definition of CM, which we have now elaborated in response to Point 6 and the histopathological definition. We have clarified this in the body of the text and I have updated to use cerebral parasite sequestration as the term for histopathologically-proven CM throughout.*

6.      Similarly, the term "WHO criteria" is used with no definition. I suspect this means a set of clinical diagnostic criteria defined by the World Health Organization, but I suggest making this much clearer up front with an appropriate reference. I also note that clinical diagnostic criteria are presented in the Methods, but it's not clear if these are the WHO criteria.

*Thanks for pointing that out. I have added the WHO definition (see page 3, line 82 - 84). These criteria, stringently applied, form the basis of the inclusion criteria. I have added a note to this effect on page 4, line 111.*

7.      Thinking on this a bit more, I'd suggest making the various ways of ascertaining MR (and their interrelationships) much clearer up front. Something like "The reference standard for ascertaining MR is by autopsy… Clinical approaches also exist, such as the WHO standard… Recent research by Taylor et al raised concerns about the WHO standard … They also found incorporating MR improved diagnostic accuracy…". It doesn't need to be a lot of words but would greatly help orient the reader.

*We have restructured the first two paragraphs to incorporate this advice. Hopefully this, in addition to addressing point 5, has made it more understandable to a general audience and specialists alike. See pages 3, line 81 – 93.*

8.      "In CM cohorts which include survivors, the proportion of those with non-malarial coma is estimated to be lower at 10%." Not sure of the relevance of this given the reference standard here is by autopsy in deceased patients. Suggest elaborating or omitting.

*Omitted, this is covered in the discussion – you are right that it is superfluous here.*

9.      "There remains a significant proportion of MR negative CM patients for whom parasitemia is a major contributor to coma, perhaps in combination with other factors, including co-infection and preexisting neurological disease" Again, while interesting I am not clear on the relevance of this in the context of this research - the authors are not attempting to ascertain these factors. Suggest elaborating or omitting.

*Omitted, this is covered in the discussion – you are right that it is superfluous here.*

10.     The "Test Methods" section of the Methods is excellent. Very precise and clear.

*Thank you! We have a lot of points to cover to improve our manuscript and including positive feedback is greatly appreciated.*

11.     Perhaps the sentence "A total of four ophthalmologist assessments were deemed to be ungradable based upon these criteria." would be more appropriately located in the Results?

*Noted, this has been moved to the Results section (see page 6, line 200 – 201).*

12.     The "Statistics" section of the methods is quite brief, with only a reference to the R package used. Suggest providing enough detail on how statistics were calculated to allow replication outside of the R package - especially calculation of 95% confidence intervals and statistical tests used to produce p-values presented. Also important to note multiplicity adjustment strategies like use of Holm method.

*testCompareR, the referenced R package, uses several methods, depending on the study population and test being conducted. We have previously published the internal mechanisms in detail and refer interested readers to that manuscript (see page 5, line 158 – 160). We feel that this provides sufficient methodological detail that analyses could be reproduced exactly, with or without the package. We have also added further methodological details for sensitivity analyses and supplementary analyses (see page 5, section Analysis).*

*If you feel the specifics of testCompareR are essential for clarity we will add them. Thanks for considering our response.*

13.     Suggest clearly articulating the hypotheses being tested before the Statistics section (and certainly before p-values in the Results).

*This is a sensible suggestion and adds clarity. Style-wise we felt this was best positioned in the Results but prior to the p values as you suggest. We have added a clear explanation on page 6, line 189 – 190 and page 6, line 199 – 199.*

14.     "MR was treated as a binary diagnostic test to predict cerebral parasite sequestration."  I understand the authors used the additional information in the ophthalmological assessments to adjust the MR positive/negative demarcation line and, consequently, improved sensitivity and specificity. Did they also consider having more than two levels of MR as the index test?

*This is an astute observation. Currently, there are no defined subclassifications of malarial retinopathy despite considerable phenotypic variability. Defining retinal phenotypes is complex and beyond the scope of this manuscript but is something we are actively working on. Watch this space!*

15.     [MINOR] Suggest including the ethics approval reference numbers.

*We need to identify these numbers from paper records. We haven’t been able to do this by the deadline for logistical reasons. To avoid any delay, we have submitted without, but we will add them at acceptance.*

16.     "There were 84 cases with retinal data." This means ~20% of your eligible participants were lost due to inadequate MR data - not a trivial proportion. Did the authors consider sensitivity analyses or other missing data approaches outside of complete case analyses? Either way, this limitation - and any potential biases arising - should be addressed in the Discussion.

*Very valid point. The challenge we faced is that imputation of the retinopathy status, when this is the key variable and there is 20% missing data, feels like guesswork and is probably not reasonable. In the case where missing data affects the index or reference tests, a sensitivity analysis assessing the worst- and best-case scenarios is appropriate (as outlined in the STARD guidelines, 2015). We have conducted these analyses. The results have been elaborated in Results (see page 6, line 213 - 222).*

17.     Suggest including an "All patients" column in Table 1.

*Done, see Table 1 (page 13).*

18.     Based on Figure 1, of the 390 patients who did not survive only 103 were included in the study. I think it is crucial to address this limitation - and any potential biases arising - in the Discussion.

*This has now been commented on in the Discussion. See page 8, line 289 – 292.*

19.     Also on Figure 1, how are the four ungradable ophthalmologist assessments incorporated into the diagram?

*This has now been indicated on the diagram. As these are a subgroup they have been added with different formatting and clear labelling to ensure this is transparent for the reader. See Figure 1 (page 14).*

20.     Currently there are elements of both methods and results in the Results on page six. I appreciate the authors are accurately presenting the sequence of events as the research progressed, but they could also consider moving some of methodological description into the Methods. While this comment is venturing into the "stylistic", more dogmatic readers may take issue with the current wording and I feel it would be remiss to not make this point.

*We feel that this enhances readability by highlighting the sequence of events, as you point out. Would you kindly consider this a minor point and allow us to maintain this as is? If either you, another reviewer or the editorial team feel this is a necessary alteration then we would, of course, be happy to comply.*

21.     Not sure of the value of including "Null hypothesis: PLR1 = PLR2 & NLR1 = NLR2, see Supplementary Methods." in the notes accompanying Table 3, as it's not readily understandable with reference to the table only. Suggest elaborating or omitting.

*Completely correct, this was left there in error. See Table 3 (page 15).*

22.      "These results indicate that MR can accurately predict cerebral sequestration in comatose children, but also indicate that the specificity of MR can be improved by a definition that excludes cases that have only 1-5 hemorrhages in a single eye." Perhaps this sentence would be more at home in the Discussion?

*This has now been moved to the first paragraph of the discussion and incorporated into the result of the test (page 7, line 224 – 240).*

23.     The Discussion is quite long compared to the rest of the paper and, in some cases, reads more like a literature review. I would suggest a heavy edit of paragraphs two, three, and four of the Discussion down to a single paragraph that concisely answers the question posed at the beginning of the first paragraph: "if surviving MR negative children do not have CM, what do they have?".

*We have compressed the first two paragraphs following the question into a single paragraph. We have maintained the third (now second) paragraph, because we felt that this sets the autopsy findings in the context of the available literature. We hope you agree that this is now more relevant (page 7 – 8, line 250 – 276).*

24.     Conversely, the Discussion is lacking a sufficiently comprehensive interpretation and contextualisation of the results presented. How do the sensitivity and specificity results achieved compare to other approaches and studies? Are there accepted standards in this (or a similar) clinical context? PPV and NPV results are presented but never discussed. *Have added this.* Are they important in this context? Why/why not? What about the approach to data missingness? Complete case analyses can introduce bias in some contexts. If this is this an issue here, should future research be conducted to investigate? What can be done to improve accuracy of diagnoses and patient outcomes given the presumably resource-constrained environment? I pose these questions not as an exhaustive list or mandatory items to cover, rather I hope they provide useful prompts for the authors to consider. Note these are in addition to the items already raised for inclusion in the Discussion.

*This was a useful criticism. Thank you. We have now indicated how results compare to only other similar study (page 7, line 232 – 233).. Along with another reviewer comment, this criticism has prompted us to define and visualise a standard workflow suitable for both well-resourced and resource-limited settings (page 8, line 275 – 276 & Figure 2). We have addressed the missing data in the Methods (page 5, line 164 – 166) and Results (page 6 – 7, line 213 - 222). We have also suggested important future research to further validate our findings (page 7, line 242 – 245). We think the discussion is much more relevant to our data now, so thank you for this feedback.*

25.     "Additionally, we were unable to compare the original cohort to the more recent cohort directly as, 20 years after the original analysis, some data were irretrievable." Apologies, I don't understand what you are saying here. You obviously have information on the original cohort in the earlier publication. Or do you mean you wanted to do a pooled analysis and needed the individual patient data? Suggest clarifying.

*This has been clarified on page 8, lines 294 – 296.*

26.     [MINOR] "In our experience, the interrater reliability between ophthalmologists and trained clinicians in detecting malarial retinopathy is good." A citation would be helpful here if available.

*This has been evaluated using stored images and has now been detailed in S6 Analysis & mentioned at page 9, line 327 - 328.*

**Reviewer #2:**

This manuscript by Wilson and colleagues is well written and of clear relevance to the malaria research and healthcare community. It re-examines the diagnostic accuracy of malarial retinopathy as an indicator of cerebral parasite sequestration in a pediatric cohort of fatal cerebral malaria (CM) cases, and proposes reclassifying patients with 1-5 hemorrhages in only one eye as retinopathy-negative. The study is well designed, the results are clearly presented, and the discussion is strong. I have only a few minor suggestions to improve clarity and impact:

1. Please adjust the language in both the abstract and main text to specify that the diagnostic utility of malarial retinopathy pertains to pediatric CM only. Several studies in adults have shown no consistent association between CM and malarial retinopathy.

*Thank you. You are absolutely right and this oversight has now been corrected. See title and abstract.*

2. Given that (i) the prevalence of anemia in Malawi exceeds the East African average (55.56%), (ii) anemia can cause anemic retinopathy, (iii) it frequently co-occurs with CM in children, and (iv) the proposed reclassification did not affect the diagnosis of anemia (Table 4), I recommend adding hemoglobin levels to Table 1. It would also be helpful to indicate how many fatal cases had concurrent anemia and to briefly discuss anemia as a potential, albeit likely infrequent, confounding factor in the limitations section.

*Excellent suggestion. This has allowed us to show that while anaemia is a possible contributor to false positives, this is relatively unlikely. This is an important consideration. We have added to the discussion to be clear that this may still affect CM + SMA kids, although this is clearly slightly different from COC + asymptomatic parasitaemia. Teasing these complex multiple pathologies apart is a challenge. We have added a reference to a recent article to this effect. See page 13, Table 1 and page 9, line 311 – 321.*

3. Considering this group's prior work and the common use of "MR" to refer to magnetic resonance, I found the use of the acronym for malarial retinopathy potentially confusing. Consider using the full term or a more distinct abbreviation to avoid ambiguity.

*This is reasonable. We have altered the text to use the full term throughout. This is probably most appropriate for an audience comprising mostly non-ophthalmologists.*

**Reviewer #3:**

The authors conducted a valuable retrospective analysis of 84 paediatric autopsy cases from the Blantyre cohort (1996-2010; page 3) to benchmark the diagnostic accuracy of malarial retinopathy (MR) against histopathologically confirmed cerebral sequestration. Among 65 WHO‐defined cerebral malaria (CM) cases, the standard MR assessment achieved sensitivity 89.4% (95% CI 77.6-95.6) and specificity 73.0% (57.2-84.8) (Table 3, page 7). By reclassifying patients with only 1-5 unilateral haemorrhages as MR‐negative, the authors report a rise in specificity to 88.0% (70.4-96.2) while sensitivity improved to 94.3% (81.7-98.7) (Table 3). This simple adjustment, if robust (see comments on methodology below), could markedly reduce misclassification of non‐CM cases and improve patient triage in resource‐limited settings.

Strengths
1. Gold‐standard reference. The use of histologically confirmed brain sequestration in 65 CM cases (page 6, Fig 1) ensures that sensitivity and specificity estimates rest on the true biological gold standard rather than imperfect clinical surrogates. This lends rare and undeniable rigor to the findings.

2. Immediate clinical relevance. A single, low‐cost rule change (1-5 haemorrhages → MR-negative) yields a clinically meaningful 12% gain in specificity without sacrificing sensitivity. In high‐burden clinics, this precision could translate into hundreds of accurately diagnosed children each year.

3. Detailed data validation. Supplementary Methods (pages 21-32) describe exhaustive chart reviews and database audits culminating in a carefully curated MR data set, demonstrating the authors' commitment to data integrity.

Major concerns

1.Blinding and observer bias: Examiners performed fundoscopy unblinded to clinical presentation (Methods, page 4, lines 12-14). Knowledge of a child's coma severity or parasitaemia can subconsciously influence grading of subtle retinal findings. Without blinded re‐grading, we cannot rule out systematic inflation of diagnostic accuracy. Recommendation: A masked, independent re‐grading of stored retinal images by at least two readers (with κ statistics) is essential to confirm that the 1-5 haemorrhage threshold truly distinguishes CM from non-CM, rather than reflecting reviewer expectations.

*This is a very fair and reasonable request, but it presents some specific challenges I want to elaborate before I explain the steps we have taken to try to comply. Firstly, this research took place in a resource limited setting. Until 2006, there was no facility to routinely image the fundi of comatose children with CM. Further, children who die of CM (and many of the controls) die very shortly after admission. Retinal photography would not usually be performed until the participant had been adequately stabilised because of the technical challenges of performing this test in patients who are unable to cooperate due to sickness. As a result, indirect fundoscopy was performed in 84 of 103 children, but fewer than 10 had retinal images stored.
Additionally, retinal imaging is not adequate for grading MR because of limitations in imaging the peripheries. This can be partially overcome using montaging techniques, in which several images are stitched together to try and get a fuller picture of the retina.*

*We have performed dual grading on retinal images from 80 eyes from 41 patients with graded retinal exams to show interrater reliability and concordance between the graded examinations and the stored images. This shows that grading of retinal images generally accords well with clinical grading via ophthalmoscopy, but results in systematic under-grading of haemorrhages. We have added commentary to this effect in the discussion. Great point – hopefully we have now shown that, although imperfect, clinical retinal examination is the gold standard for malarial retinopathy assessment. This analysis is available in S6 Analysis and has been referenced in the text on page 9, line 303 – 309.*

2. Spectrum bias and generalisability: The cohort comprises only fatal CM cases (Fig 1, page 6), likely representing the most severe end of the disease spectrum. Retinal findings may be more pronounced in children who die, artificially boosting sensitivity and specificity. When applied to survivors or less severe cases, the refined criterion could underperform, leading to missed diagnoses or false reassurance. Recommendation: Either incorporate a non-fatal CM cohort or model the expected shift in test metrics (e.g. using the difference in median time to death between MR-/no sequestration and MR-/sequestration groups in Table 1, page 5) to demonstrate that the 1-5 haemorrhage cutoff will hold true across the full clinical spectrum.

*This recommendation is very helpful. Because no suitable validation cohort exists (see response to point 6), we have modelled the effects of various clinical variables using a logistic regression model with LASSO regularisation. This model suppressed all clinical variables and their interactions with the reference test to zero, suggesting that they exert little influence on the result of the reference or index tests. See page 7, line 217 - 222 and S3 Analysis.*

3. Verification bias and missing data: 19 of 103 autopsied children lacked MR exams and were excluded (Fig 1, page 6), yet no comparison of baseline characteristics was presented. If excluded children systematically differed (for instance, younger age or lower parasitaemia), the retained sample may not be representative, skewing accuracy estimates. Recommendation: Compare age, parasitaemia, Blantyre Coma Score and other key variables between included and excluded patients (drawing from Table 1 data). Perform multiple imputation or sensitivity analyses to show that the 1-5 haemorrhage rule remains robust even when accounting for missing examinations.

*These are excellent suggestions, thank you. We have now reported the differences between those with missing data and those included in the study. Sensitivity analyses using extreme case methodology have also been conducted to give clarity on the results. In conjunction with your suggestion above, we can infer that the differences between the cohorts are unlikely to have influenced the results. This has been reported in both the Results (page 6 – 7, line 213 – 217) and Discussion (page 7, line 228 – 245), as well as S5 Table.*

4. Statistical precision and additional metrics: Table 3 omits positive and negative likelihood ratios (LR+ and LR-) and does not provide confidence intervals for the 12% specificity increase. Likelihood ratios translate test results into changes in disease probability at the bedside, directly informing clinical decisions. Confidence intervals for Δspecificity quantify uncertainty around the observed gain and guard against over-interpretation of chance findings. Recommendation: Calculate LR+ and LR- with 95% CIs (e.g., original PLR ~3.3 [2.0-5.7], recoded ~7.9 [3.1-19.0]) and report the CI for the specificity difference to demonstrate that the improvement is both clinically and statistically meaningful.

*This suggestion improves the standard of reporting. It also prompted us to reflect on our presentation of these results in the discussion and the significance has now been highlighted. See page 6, line 209 and Table 3, page 16.*

5. Borderline p-value interpretation and power: The specificity improvement yields p = 0.065 (Table 3, page 7), narrowly missing α = 0.05. Presenting a p value alone without context risks dichotomous thinking. A suggestive trend may nonetheless hold clinical importance, but without a post-hoc power analysis or confidence interval for Δspecificity, readers cannot assess whether the study was simply underpowered. Recommendation: Reframe p = 0.065 as a compelling trend, present the confidence interval for the specificity gain, and include a post-hoc power calculation (e.g., with n = 60, α = 0.05, the study has ≥80% power to detect a 15% specificity difference) to contextualize the finding's reliability.

*We have rephrased to identify the compelling nature of the trend and we have conducted post-hoc power calculations (via simulation) and added to the Results. See rewrite of Discussion (page 7, line 236 - 245) and Results (page 6, line 209 - 212).*

6. Risk of over-fitting and need for external validation: The 1-5 haemorrhage threshold was both derived and tested within the same small subset (n = 60), raising the possibility of over-fitting. Without independent validation, there is a high risk that the cutoff works well only in this sample, limiting broader adoption and potentially leading to clinical misclassification elsewhere. Recommendation: Validate the refined threshold prospectively in an independent cohort—such as survivors from Beare et al. 2006 or a multi-centre study — with pre-specified performance targets and formal sample-size justification to confirm generalisability.

*This is very challenging. Your criticism is both astute and valid. However, so far as we are aware there are no comparable cohorts that include both sequestration data from paediatric autopsy and graded fundoscopy data. There is no definitive ground truth for CM diagnosis CM survivors and current models are either insufficiently sensitive and specific to be used as the reference test (Watson et al.) or they include retinopathy as a predictor (Lin et al.).*

*You are right to point out that what is really needed is a large, multicentre autopsy study, probably using minimally invasive sampling methods, which includes MR data collected during life. As well as providing a validation data set, this would provide a dataset from which models could be derived that are sufficiently accurate to include survivor cohorts. We have added this important point to the discussion (see page 7, line 242 - 245).*

Statistical analysis and reporting

1. Threshold derivation transparency: Clarify whether the 1-5 haemorrhage cutoff was pre-specified based on prior data or emerged from exploratory analyses. If the latter, describe corrections for multiple testing to safeguard against Type I error.

*This cut-off was determined before any hypothesis testing but following chart review. This has been clarified in the body of the text. See Results (page 6, line 196 - 199).*

2. ROC curve analysis: An ROC analysis of haemorrhage count (0 vs 1-5 vs >5) would yield an AUC, providing an objective measure of overall discrimination and reinforcing the choice of cutoff.

*ROC curves are, by their nature, univariate. Here, we are using a multivariate definition of malarial retinopathy and the change to the cut off for haemorrhages adjusts this multivariate definition. This is not easily visualised. Plotting the ROC curve for a single variable (haemorrhages) is unlikely to provide much additional information as we have shown previously that haemorrhages alone are poorly predictive of sequestration. We have mentioned this in the discussion (page 7, line 236 - 239). It is also clear from this point, and some below, that the phrasing of the definition change is unclear. This has now been adjusted throughout the manuscript (a good example of this language change is on page 9, line 336 – 338).*

3. Handling of missing data: Specify the exact number and reasons for MR or sequestration data omissions; describe any imputation methods or decision rules applied to ensure complete transparency.

*Insofar as this is possible with our retrospective data, we have complied with this suggestion (see page 6, line 184 - 188). Thanks for pointing this out.*

Presentation and clarity

1. Illustrative fundus images: Include high-quality examples of (a) 1-5 unilateral haemorrhages, (b) >5 bilateral haemorrhages, and (c) whitening/vessel changes, to guide clinicians in real-world grading.

*Members of our group have done this previously. We agree, though, that this is useful and we have directed interested readers to the previous paper (see page 4, line 129 - 130).*

2. Practical implementation tools: Provide a concise, step-by-step flowchart or checklist for non-ophthalmologist clinicians, enhancing consistent application of the refined criterion in busy, resource-limited wards.

*This is a great idea. It is clear that we have not adequately explained the classification change based on haemorrhages, nor really considered its relevance in view of the literature and the steps a clinician should take. We think the addition of the flow chart as suggested clarifies this (see Figure 2, page 18). Thank you.*

3. Comprehensive integration of MR features: Discuss how the refined haemorrhage rule complements other MR signs—retinal whitening and vessel changes—in a combined diagnostic algorithm, maximizing overall accuracy.

*The definition of MR is multivariate. This has hopefully now been clarified by responding to your comment about the ROC curve. Apologies for the confusion and thank you for the constructive feedback.*

Recommendation
Major revision. Addressing these methodological, statistical, and presentation enhancements will transform a promising retrospective finding into a robust, generalisable diagnostic tool, ultimately improving care and outcomes for children with suspected cerebral malaria in endemic regions.

Any attachments provided with reviews can be seen via the following link:

--------------------------------------------------------- ---
General editorial requests:

(Note: not all will apply to your paper, but please check each item carefully)

*       We ask every co-author listed on the manuscript to fill in a contributing author statement, making sure to declare all competing interests. If any of the co-authors have not filled in the statement, we will remind them to do so when the paper is revised. If all statements are not completed in a timely fashion this could hold up the re-review process. If new competing interests are declared later in the revision process, this may also hold up the submission. Should there be a problem getting one of your co-authors to fill in a statement we will be in contact. Please do not add or remove authors without first discussing this with the handling editor. You can see our competing interests policy here: <http://journals.plos.org/plosmedicine/s/competing-interests>.

*I will contact all co-authors to facilitate this process.*

*       Please upload any figures associated with your paper as individual TIF or EPS files with 300dpi resolution at resubmission; please read our figure guidelines for more information on our requirements: <http://journals.plos.org/plosmedicine/s/figures>. While revising your submission, please upload your figure files to the PACE digital diagnostic tool, <https://pacev2.apexcovantage.com/>. PACE helps ensure that figures meet PLOS requirements. To use PACE, you must first register as a user. Then, login and navigate to the UPLOAD tab, where you will find detailed instructions on how to use the tool. If you encounter any issues or have any questions when using PACE, please email us at [PLOSMedicine@plos.org](mailto:PLOSMedicine@plos.org).

*Uploaded images have all been passed through PACE. There are 3 figures, total. Figure1, Figure2 and S5_Figure. All are provided as TIF files.*

*       Please ensure that the paper adheres to the PLOS Data Availability Policy (see <http://journals.plos.org/plosmedicine/s/data-availability)>, which requires that all data underlying the study's findings be provided in a repository or as Supporting Information. For data residing with a third party, authors are required to provide instructions with contact information (web or email address) for obtaining the data. Please note that a study author cannot be the contact person for the data. PLOS journals do not allow statements supported by "data not shown" or "unpublished results." For such statements, authors must provide supporting data or cite public sources that include it.

*Done.*

*       We expect all researchers with submissions to PLOS in which author-generated code underpins the findings in the manuscript to make all author-generated code available without restrictions upon publication of the work. In cases where code is central to the manuscript, we may require the code to be made available as a condition of publication. Authors are responsible for ensuring that the code is reusable and well documented. Please make any custom code available, either as part of your data deposition or as a supplementary file. Please add a sentence to your data availability statement regarding any code used in the study, e.g. "The code used in the analysis is available from Github [URL] and archived in Zenodo [DOI link]" Please review our guidelines at <https://journals.plos.org/plosmedicine/s/materials-software-and-code-sharing> and ensure that your code is shared in a way that follows best practice and facilitates reproducibility and reuse. Because Github depositions can
be readily changed or deleted, we encourage you to make a permanent DOI'd copy (e.g. in Zenodo) and provide the URL.

*Data and code are available. Github repo will be made public and archived at the point of acceptance. Thank you!*


* In you ethics statement, Please include IRB approval numbers.

*We need to identify these numbers from paper records. We haven’t been able to do this by the deadline for logistical reasons. To avoid any delay, we have submitted without, but we will add them at acceptance.*

*       Please ensure that the study is reported according to the STARD guideline and include the completed STARD checklist as Supporting Information. When completing the checklist, please use section and paragraph numbers, rather than page numbers. Please add the following statement, or similar, to the Methods: "This study is reported as per STARD guideline (S1 Checklist)."

*Done*

FORMATTING - GENERAL
*       Abstract: Please structure your abstract using the PLOS Medicine headings (Background, Methods and Findings, Conclusions). Please combine the Methods and Findings sections into one section.

*Done – please note, this contradicts slightly the later instruction to use STARD for Abstracts. I have tried to accommodate both bits of guidance.*

*       At this stage, we ask that you include a short, non-technical Author Summary of your research to make findings accessible to a wide audience that includes both scientists and non-scientists. The Author Summary should immediately follow the Abstract in your revised manuscript. This text is subject to editorial change and should be distinct from the scientific abstract. Ideally each sub-heading should contain 2-3 single sentence, concise bullet points containing the most salient points from your study. In the final bullet point of 'What Do These Findings Mean?', please include the main limitations of the study in non-technical language. Please see our author guidelines for more information: <https://journals.plos.org/plosmedicine/s/revising-your-manuscript#loc-author-summary>.

*Done, see page 2, line 51.*

*       Please express the main results with 95% CIs as well as p values. When reporting p values please report as p<0.001 and where higher as the exact p value p=0.002, for example. Throughout, suggest reporting statistical information as follows to improve clarity for the reader "22% (95% CI [13%,28%]; p</=)". Please be sure to define all numerical values at first use

*Done*.

*       Please include page numbers and line numbers in the manuscript file. Use continuous line numbers (do not restart the numbering on each page).

*Done*

*       Please cite the reference numbers in square brackets. Citations should precede punctuation.

*Done*

FIGURES AND TABLES
*       Please provide titles and legends for all figures and tables (including those in Supporting Information files).

*Done. These have been provided under ‘Supplementary Information’. Page 11, line 417.*


SUPPLEMENTARY MATERIAL
*       Please note that supplementary material will be posted as supplied by the authors. Therefore, please amend it according to the relevant comments outlined here.

*       Please cite your Supporting Information as outlined here: <https://journals.plos.org/plosmedicine/s/supporting-information>

REFERENCES
*       PLOS uses the numbered citation (citation-sequence) method and first six authors, et al.
*Done according to PLOS Medicine template from Zotero.*

*       Please ensure that journal name abbreviations match those found in the National Center for Biotechnology Information (NCBI) databases (<http://www.ncbi.nlm.nih.gov/nlmcatalog/journals>), and are appropriately formatted and capitalised.

*Done*

*       Where website addresses are cited, please include the complete URL and specify the date of access (e.g. [accessed: 12/06/2023]).

*       Please also see <https://journals.plos.org/plosmedicine/s/submission-guidelines#loc-references> for further details on reference formatting.

DIAGNOSTIC STUDIES

*       Please ensure that the study is reported according to the STARD guideline (<https://www.equator-network.org/reporting-guidelines/stard/>) and include the completed STARD checklist as Supporting Information. Please add the following statement, or similar, to the Methods: "This study is reported as per the Standards for Reporting of Diagnostic Accuracy (STARD) guideline (S1 Checklist)." When completing the checklist, please use section and paragraph numbers, rather than page numbers.

*Done*

*       Please structure your Abstract according to STARD for Abstracts (<https://www.equator-network.org/reporting-guidelines/stard-abstracts/>).

*See previous comment on abstract*

*       Please structure the Methods section using the following sub-headings: Study design, Participants, Test methods, Analysis.

*Done, with additional “Ethics”. This could be moved to the end at the editorial team’s request.*

*       Please include a diagram to describe the flow of participants through the study (typically figure 1).

*Done*
