# Supplementary material for: Re-evaluating malarial retinopathy to improve its diagnostic accuracy in paediatric cerebral malaria: A retrospective study
Source: PLoS Med. 2025 Sep 15;22(9):e1004727. doi: 10.1371/journal.pmed.1004727 (PMC12445540; doi:10.1371/journal.pmed.1004727)
Supplement: Attachment — Submitted filename: 20250804_MRSens_ReviewerResponses_v2.0.docx [file pmed.1004727.s015.docx]

We thank the reviewers for re-reviewing this manuscript after having provided the detailed feedback. Find below point-by-point response addressing the editorial and reviewer comments outstanding following this second review.

We look forward to sharing our improved manuscript with the scientific community. Thank you for your time and effort!

**Comments from the academic editor:**
We kindly ask you to remove the power calculation (which was requested by R3, concern #5.), as post-hoc power calculations are considered bad statistical practice. Please accept my apologies for not catching this in the previous round of review, and the extra work that this has caused.

*Done. No problem. It might be useful for planning future work, so no time was wasted.*

Please address the remaining suggestions from Reviewer #1.
*See Reviewer 1 comments below.*

Table 3: please specifcy the size of the confidence interval. is this 95%?
*I have added this for clarity. They are indeed 95% CIs. Thanks.*

Please remove the second period at the end of your Background section.
*Done.*

We kindly ask you at this stage to include the URLs for Github and Zenodo.
*Done.*

Are the MP_numbers in the supplemental data identifiers of the patients? Please review our guidance on patient confidentiality:
It appears that you have included data that may breach patient confidentiality. Please edit your data set in accordance with patient consent, and remove any identifying data.

*I fully anonymised all data before uploading the manuscript. I also noted this in the Supplementary Information section towards the end. However, the numbers do look like real numbers. They are ‘MP’ (Malaria Project), ‘a’ (anonymous) and three digits (randomly assigned numbers). I have removed all data and reuploaded to ensure that we are indeed using the anonymised source.*

*can you clarify what supplemental information “32c2c9f8-42d8-446b-96fc-dfcb0b49de22” and “1c7a87b1-5594-4c02-a605-98c0175fd634” are?

*They are .RData files. Including them would allow users to run any of the analyses themselves after downloading the data and scripts. However, to avoid any conflicts caused by unusual file types I have just added a README.md file to the Github repository explaining the order in which the analyses should be run. These files will be generated locally when a user runs the script and can now be safely removed from the supplementary information. For more clarity, feel free to check the instructions on Github.*

**Comments from the reviewers:**

**Reviewer #1:**

The authors have addressed all my comments, and I am happy to recommend acceptance. I have included a few suggestions/notes below (numbers reference my original review items), but these are entirely optional. Well done and congratulations.

4. Suggest also using an alternate word for "significant" in the first sentence of the Discussion (line 228).

*Have changed to notable. Thanks.*
6. Thank you - this is much clearer. I suggest also including a citation for the WHO criteria when first discussed at line 82.

*Done.*
20. My apologies - I should have flagged this as minor. I am OK with the authors' proposal.

*Thanks.*
24. The revised Discussion is clear, cogent, and comprehensive.

*Thank you.*

**Reviewer #2:**

The authors have adequately addressed all my concerns in the edited manuscript.

*Thank you.*

**GENERAL EDITORIAL REQUESTS**

* Please confirm that your title complies with to PLOS Medicine's style. Your title must be nondeclarative and not a question. It should begin with main concept if possible. "Effect of" should be used only if causality can be inferred, i.e., for an RCT. Please place the study design ("A randomized controlled trial," "A retrospective study," "A modelling study," etc.) in the subtitle (ie, after a colon).

*Have updated accordingly.*

* Please confirm that your abstract complies with our requirements, including format (three sections: Background, Methods and Findings, and Conclusions) and providing all the information relevant to this study type <https://journals.plos.org/plosmedicine/s/submission-guidelines#loc-abstract>

*Have added a couple of lines to ensure adherence to the PLOS Medicine standard.*

* Please ensure that the Introduction ends with a clear description of the study question or hypothesis.

*Added a few words to state the hypothesis explicitly.*

* Please ensure that all abbreviations are defined at first use throughout the text.

* Please confirm that all numbers presented in the abstract are present and identical to numbers presented in the main manuscript text.

*Have updated the numbers in the abstract.*

* Please confirm that all original data are available in this manuscript. If this is not the case, please specify Whether these data can be obtained:

  a) If the data are freely or publicly available, note this and state the location of the data: within the paper, in Supporting Information files, or in a public repository (include the DOI or accession number).

  b) If the data are owned by a third party but freely available upon request, please note this and state the owner of the data set and contact information for data requests (web or email address). Note that a study author cannot be the contact person for the data.

  c) If the data are not freely available, please describe briefly the ethical, legal, or contractual restriction that prevents you from sharing it. Please also include an appropriate contact (web or email address) for inquiries (again, this cannot be a study author).

*Confirmed. This is fully anonymised and is stated in the Data availability section.*

GENERAL
In the last sentence of the Abstract Methods and Findings section, please describe the main limitation(s) of the study's methodology.

*Done.*

FUNDING STATEMENT

The funding statement should include: specific grant numbers, initials of authors who received each award, URLs to sponsors’ websites. Also, please state whether any sponsors or funders (other than the named authors) played any role in study design, data collection and analysis, the decision to publish, or preparation of the manuscript. If they had no role in the research, include this sentence: “The funders had no role in study design, data collection and analysis, decision to publish, or preparation of the manuscript.”

*I have added this line.*

It appears that one or more study authors is affiliated with one or more of the agencies that funded the study. Thus, the statement “The funders had no role in study design, data collection and analysis, decision to publish, or preparation of the manuscript” does not apply. Please revise the Financial Disclosure accordingly, as in "[Author name] is [author's role] at [funding agency]. The funders had no other role in study design…..”

*This was added as a comment in error and has been discussed with Dr. Suzanne De Bruijn. It is considered resolved.*

COMPETING INTERESTS STATEMENT

All authors must declare their relevant competing interests per the PLOS policy, which can be seen here: <https://journals.plos.org/plosmedicine/s/competing-interests> For authors with ties to industry, please indicate whether any of the interests has a financial stake in the results of the current study.

*See above comment. Thank you.*

ETHICS AND CONSENT

At this time, please provide the name(s) of the institutional review board(s) that provided ethical approval, as well as the approval numbers.

*Done.*

FIGURES
Please provide titles and legends for all figures and tables (including those in Supporting Information files). Please define all acronyms used in each figure or table in its corresponding legend.

*I have added in-line legends to the tables throughout the supplementary information. I have also provided captions for the supplements at the very end of the manuscript (see ). I hope this meets requirements. Thank you!*

DIAGNOSTIC TESTS

Did your study have a prospective protocol or analysis plan? Please state this (either way) early in the Methods section.

a) If a prospective analysis plan (from your funding proposal, IRB or other ethics committee submission, study protocol, or other planning document written before analyzing the data) was used in designing the study, please include the relevant prospectively written document with your revised manuscript as a Supporting Information file to be published alongside your study, and cite it in the Methods section. A legend for this file should be included at the end of your manuscript.

b) If no such document exists, please make sure that the Methods section transparently describes when analyses were planned, and when/why any data-driven changes to analyses took place.

c) In either case, changes in the analysis-- including those made in response to peer review comments-- should be identified as such in the Methods section of the paper, with rationale.

*I have amended the text in the results and methods to make clear the point at which a new hypothesis was formulated. Thank you!*

For all observational studies, in the manuscript text, please indicate: (1) the specific hypotheses you intended to test, (2) the analytical methods by which you planned to test them, (3) the analyses you actually performed, and (4) when reported analyses differ from those that were planned, transparent explanations for differences that affect the reliability of the study's results. If a reported analysis was performed based on an interesting but unanticipated pattern in the data, please be clear that the analysis was data-driven.

*I believe in responding to the previous comments I have now also conformed to this guidance. See previous comments and*
